# Factors associated with myopia in 19-year-old adult men in Korea between 2014 and 2020

**DOI:** 10.1038/s41598-023-38569-w

**Published:** 2023-07-18

**Authors:** So Hyeon Gwon, Dong Cheol Lee

**Affiliations:** 1 Department of Ophthalmology, School of Medicine, Keimyung University, Daegu, Republic of Korea; 2 Department of Ophthalmology, Dongsan Medical Center, Keimyung University School of Medicine, #1035, Dalgubeol-daero, Dalseo-gu, Daegu, 42601 Republic of Korea

**Keywords:** Diseases, Medical research, Risk factors

## Abstract

Numerous environmental factors that influence myopia have been studied, but only few factors have been definitively identified. We examined factors influencing myopia using data from 2014 to 2020 physical examinations received from the Korean Military Manpower Administration. We used the Cochran–Armitage trend test to investigate the annual prevalence of myopia and high myopia. To determine risk factors for myopia, logistic regression was performed. The data of 2,215,126 19-year-old Korean men were examined. The myopia and high myopia prevalences showed significant annual increases; in 2020, these prevalences were 58.9% and 18.0%, respectively. The myopia prevalence was high when the birth season was spring, education level was high, height was small, weight and body mass index (BMI) were low (< 18.5 kg/m^2^), and color vision was normal (all, p < 0.05). The high myopia prevalence was high when the birth season was spring, education level was high, height was tall, weight and BMI were low (< 18.5 kg/m^2^), and color vision was normal (all, p < 0.05). The prevalence of myopia and high myopia in this population is increasing annually. The risk of both conditions increased when the birth season was spring, education level was high, BMI was low, color vision was normal, and diabetes was present.

## Introduction

Globally, the “myopia epidemic” is a recent occurrence and is often found among young people. Currently, the prevalence of myopia and high myopia among young people can lead to complications as they age. Visual impairment is projected to increase by 2050, which may be a result of the combined effects of increased myopia, mean spherical equivalent (SE), axial length change, and aging^1^. Recently, up to 90% of teenagers and young adults have myopia, and especially in Seoul, South Korea, 96.5% of 19-year-old men have myopia^2,3^. The prevalence of myopia in Asia is higher than that in the West^4^, and Korea has a higher myopia prevalence than other Asian countries^5^. In a previous study on young Korean men, the prevalences of myopia and high myopia were increasing annually^6^. According to a Chinese study, the rate of myopia increased each year, and the age at which the refractive error became myopic and stabilized was at 20 years^7^.

The causes of myopia are mostly genetic and environmental factors. Factors related to myopia include obesity^3,8,9^, high educational level^3,10^, residential area^4^, birth season^10,11^, parental myopia^12,13^, and time spent outdoors^14–16^. A previous study of a rural Korean population^9^ reported no association of myopia with color vision. On the other hand, other studies found a lower prevalence of myopia based on color vision deficiency^17–19^. A study of children with diabetes^12^ found no significant association between diabetes and myopia, although the prevalence of myopia may increase transiently in patients with diabetes at a younger age. However, a study in Taiwan^20^ found a higher prevalence of myopia in the diabetic group than in the control group, but no significant difference was noted in high myopia prevalence. However, only few genetic and environmental factors have been definitively identified. In addition, population-based studies with high response rates, sufficient population size, and few biases provide robust evidence for determining the etiology of myopia.

In this study, among the aforementioned factors affecting myopia, data obtained from annual physical examinations according to the Korean conscription regulations were used. Bias was reduced by limiting the prevalence of myopia to only 19-year-old men for 7 years, from 2014 to 2020, and a more scientific approach was possible with approximately 300,000 people included per year. Based on this, we aimed to investigate the trends in the prevalences of myopia and high myopia and the factors that may affect these conditions (season of birth, education level, height, weight, body mass index [BMI], color vision, and diabetes) with less bias compared to other previous papers.

## Methods

### Ethics statements

This study was approved by Keimyung University Dongsan Hospital Institutional Review Board (DSMC 2021-11-071-005), and all methods were performed in accordance with the relevant guidelines and regulations. The Keimyung University Dongsan Hospital Institutional Review Board waived the requirement for informed consent, as this study used existing data that were de-identified.

### Participants and study design

The data were provided by the Military Manpower Administration (MMA) of the Republic of Korea. In Korea, adult men aged > 18 years are required to join the military, before which they undergo mandatory physical examination. Most of them undergo the same physical examination at the MMA at 19 years of age. In this study, only men aged 19 years were included. Those who received duplicate examinations and cases of missing physical examinations were excluded. Thus, a total of 2,215,126 men were analyzed.

Data were collected from the MMA in Korea. Because the database contained personal information, the MMA provided de-identified data. Data preprocessing, categorization, and statistical analysis were conducted by the Korea Data Agency (K-data, Seoul, Korea).

### Physical examination

When visual acuity was < 0.3 without glasses, an automatic refraction tester (RF 10, Canon Inc., Tokyo, Japan) was used. We used the Korean color vision test book to assess color vision (*Han Cheon-Seok Hansik Color Blind Test Book*, Seoul, Korea). Height was measured in cm, and weight was measured in kg. For the blood glucose test, fasting was recommended from the evening of the previous day, and urine samples were analyzed on the day of the test. Glucose levels were measured using blood and urine samples. Urine tests were performed using URiSCAM Super + (YD Diagnostics, Yongin, Korea) and AX4030 (Arkray, Kyoto, Japan). For the blood glucose test, AU5811 and AU5810 (Beckman Coulter Inc., Brea, CA, USA) and COBAS, C702 (Roche, Basel, Switzerland) were used. The inspection machine may have been slightly different for each regional office.

### Definitions

Men were included in the myopia group if at least one of their eyes had myopia. For men with myopia in both eyes, we examined data for the eye with higher myopia. The definition criteria were divided as follows with reference to a previous study^6^. Refraction measurements were converted into the SE, calculated as the spherical value plus half of the astigmatic value (sphere + cylinders/2). When SE was < − 0.5, the condition was classified as myopia, and when it was < − 6.0, the condition was classified as high myopia. BMI was calculated by dividing the weight (kg) by the height squared (m^2^).

The birth season was divided into spring (March–May), summer (June–August), autumn (September–November), and winter (December–February). Education level was divided into less than high school, 2–3-year university, and 4-year university or higher. Height was categorized as ≤ 170, 171–174, 175–178, and ≥ 179 cm; weight was categorized as ≤ 60, 61–66, 67–75, and ≥ 76 kg; and BMI was categorized as < 18.5, 18.5–22.99, 23.00–24.99, and ≥ 25 kg/m^2^. Additionally, color vision and diabetes were used based on the categories provided by the MMA. Color vision was classified as normal, color weakness, and color blindness; and diabetes was classified as present or absent.

### Statistical analyses

Continuous variables were analyzed using the t-test and are expressed as mean and standard deviation. Categorical variables were analyzed using the chi-square test and are expressed as frequency and percentage. Continuous data were categorized and used to facilitate interpretation. Therefore, categorical analysis was used. From 2014 to 2020, the Cochran–Armitage trend test was conducted to investigate the trends in the prevalence of myopia and high myopia by year.

A generalized linear model was used to investigate the risk factors affecting myopia. Since the dependent variable was binomial, we created a logistic regression model. Each factor was analyzed as a univariate to determine the factors influencing the prevalence of myopia. Additionally, multivariate analysis was performed and included all variables except for height and weight.

As all men of Korean nationality undergo the same physical examination, it was judged that there would be less bias, and only those aged 19 years were selected in this study to further reduce the bias. If at least one eye had myopia, we tried to include it in the myopia group to increase the sample size. Moreover, if cases of missing information, the data of that individual were partially excluded from the analysis. Statistical analyses were performed using R, version 4.1.3 (R Project for Statistical Computing, Vienna, Austria). All statistical methods were tested at the significance level of 0.05.

## Results

### Demographic characteristics

From 2014 to 2020, a total of 2,304,552 people underwent physical examination by the MMA, 2,259,853 of whom were aged 19 years. Of these, the data of 2,215,126 individuals were included in the study, except when there were no refractive values for the left or right eye.

In total, the data of 2,215,126 men collected over 7 years from 2014 to 2020 were included. The average height, weight, and BMI were 173.64 cm, 70.46 kg, and 23.28 kg/m^2^, respectively. The proportions of participants born in spring, winter, autumn, and summer were 26%, 25.8%, 24.9%, and 23.2%, respectively. The most common education level was a 4–6-year university degree or higher. Color vision was mostly normal, and diabetes was mostly absent. There were 1,248,941 (56.4%) men with myopia and 373,351 (16.9%) with high myopia. Participants with myopia and high myopia showed significant differences in height, weight, and BMI compared with participants in the non-myopia and non-high myopia groups (all p <0.001 except high myopia BMI(p=0.022), respectively), as well as significant differences in the year, birth season, education level, and color vision (all p < 0.001). Diabetes was not associated with myopia and high myopia (p = 0.143 and p = 0.288, respectively; Table 1).

**Table 1:** Demographics of the study population.

		Total		Non-Myopia		Myopia		p-value	Non-High Myopia		High Myopia		p-value
		(n = 2,215,126)		(n = 966,185)		(n = 1,248,941)			(n = 1,841,775)		(N = 373,351)		
		Mean or Count	SD or %	Mean or Count	SD or %	Mean or Count	SD or %		Mean or Count	SD or %	Mean or Count	SD or %	
Height (Mean ± SD)		173.64	5.79	173.70	5.78	173.60	5.79	< 0.001	173.63	5.78	173.71	5.82	< 0.001
Weight (Mean ± SD)		70.46	14.51	70.77	14.37	70.22	14.61	< 0.001	70.44	14.40	70.57	15.00	< 0.001
BMI (Mean ± SD)		23.28	4.44	23.37	4.39	23.21	4.47	< 0.001	23.28	4.41	23.29	4.59	0.022
Year	2014	351,064	15.8	167,072	17.3	183,992	14.7	< 0.001	296,282	16.1	54,782	14.7	< 0.001
	2015	337,927	15.3	153,601	15.9	184,326	14.8		285,945	15.5	51,982	13.9	
	2016	327,374	14.8	139,180	14.4	188,194	15.1		272,385	14.8	54,989	14.7	
	2017	312,347	14.1	135,504	14.0	176,843	14.2		258,334	14.0	54,013	14.5	
	2018	302,736	13.7	130,005	13.5	172,731	13.8		250,586	13.6	52,150	14.0	
	2019	312,175	14.1	129,330	13.4	182,845	14.6		255,580	13.9	56,595	15.2	
	2020	271,503	12.3	111,493	11.5	160,010	12.8		222,663	12.1	48,840	13.1	
Birth season	Spring	575,583	26.0	247,919	25.7	327,664	26.2	< 0.001	476,863	25.9	98,720	26.4	< 0.001
	Summer	514,910	23.2	225,531	23.3	289,379	23.2		428,431	23.3	86,479	23.2	
	Autumn	552,631	24.9	242,245	25.1	310,386	24.9		460,527	25.0	92,104	24.7	
	Winter	572,002	25.8	250,490	25.9	321,512	25.7		475,954	25.8	96,048	25.7	
Education level	Less than high school	513,889	23.2	267,167	27.7	246,722	19.8	< 0.001	450,233	24.4	63,656	17.0	< 0.001
	2–3-year university	519,872	23.5	252,864	26.2	267,008	21.4		446,376	24.2	73,496	19.7	
	4-year university or higher	1,181,365	53.3	446,154	46.2	735,211	58.9		945,166	51.3	236,199	63.3	
Color vision	Normal	2,109,911	95.3	919,103	95.1	1,190,808	95.3	< 0.001	1,751,617	95.1	358,294	96.0	< 0.001
	Color weakness	104,972	4.7	46,972	4.9	58,000	4.6		89,929	4.9	15,043	4.0	
	Color blindness	243	0.0	110	0.0	133	0.0		229	0.0	14	0.0	
Diabetes	No	2,197,064	99.2	958,209	99.2	1,238,855	99.2	0.143	1,826,811	99.2	370,253	99.2	0.288
	Yes	18,062	0.8	7976	0.8	10,086	0.8		14,964	0.8	3098	0.8	

BMI, body mass index; SD, standard deviation.

### Prevalence of myopia and high myopia

**Figure 1:**
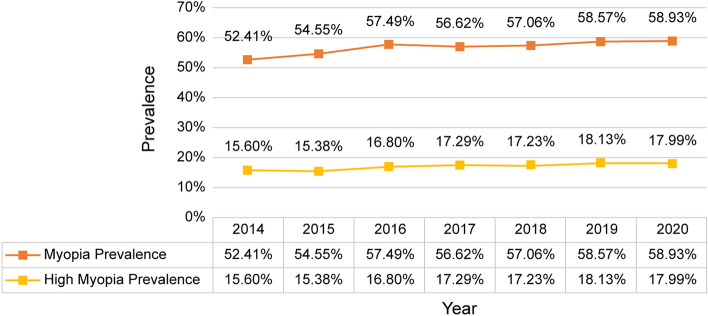
Prevalence of myopia and high myopia.

From 2014 to 2020, the prevalence of myopia was 52.4%, 54.5%, 57.5%, 56.6%, 57.1%, 58.6%, and 58.9%, respectively. From 2014 to 2020, the prevalence of high myopia was 15.6%, 15.4%, 16.8%, 17.3%, 17.2%, 18.1%, and 18.0%, respectively (Fig. 1). The Cochran–Armitage trend test was performed to statistically evaluate the prevalence of myopia and high myopia. The prevalence of myopia increased from 2014 to 2020 (p < 0.001). High myopia also showed an increasing trend (p < 0.001). Therefore, the prevalences of myopia and high myopia increased during the examined period.

### Factors affecting myopia

According to univariable logistic regression analysis, the prevalence of myopia was the highest when the birth season was spring, followed by summer and winter, and low when the birth season was autumn. Compared with spring-born individuals, individuals born in summer, fall, and winter had odds ratios of 0.971, 0.969, and 0.971 for developing myopia, respectively (p < 0.001). The level of education was higher for those who attended 2–3-year or ≥ 4-year college than for those who graduated from high school. Moreover, the prevalence of myopia was higher among men who attended 2–3-year college and lower among men who attended 4-year college than among men who graduated high school. Compared with high school graduation, a higher education level had odds ratios of 1.143 and 1.784 for myopia development (2–3-year university and ≥ 4-year university courses, respectively; p < 0.001). With regard to height, the prevalence of myopia was high among individuals with a height of ≤ 170 cm, and it was lower in the taller group than in the shorter group. Compared with the height of ≤ 170 cm, heights of 171–174, 175–178, and ≥ 179 cm were associated with odds ratios of 0.972, 0.958, and 0.944, respectively (p < 0.001). The prevalence of myopia was high when the weight was ≤ 60 kg, and it was lower in the 66–75-kg group than in the other weight groups. Compared with the ≤ 60-kg group, the odds ratios for myopia development were 0.887, 0.851, and 0.871 for the 61–66-, 67–75-, and ≥ 76-kg groups, respectively (p < 0.001). With regard to BMI, values of < 18.5 and 23–25 kg/m^2^ were associated with a high and low prevalence of myopia, respectively. The odds ratios for myopia development in the 18.5–22.99-, 23.00–24.99-, and ≥ 25-kg/m^2^ groups, compared with the < 18.5-kg/m^2^ group, were 0.808, 0.770, and 0.806, respectively (p < 0.001). The prevalence of myopia increased every year when the height, weight, and BMI were ≤ 170 cm, ≤ 60 kg, and < 18.5 kg/m^2^, respectively, and it was high when the participant had normal color vision. However, the odds ratio for myopia development was 0.953 for participants with color weakness (p < 0.001); moreover, it was negative for participants with color blindness, although this finding was not statistically significant. Color blindness and diabetes were not significantly associated with myopia.

Because BMI provides information about height and weight, we only included BMI in the multivariate analysis. The results of multivariable logistic regression analysis showed the effect of each factor on myopia after adjustment for the other factors. The odds ratios for developing myopia among participants born in summer, fall, and winter, compared with those born in spring, were 0.976, 0.971, and 0.972, respectively (p < 0.001). Compared with high school graduates, participants who completed 2–3-year college and ≥ 4-year college presented odds ratios of 1.148 and 1.804, respectively (p < 0.001). The odds ratios for the greater BMI groups, compared with the < 18.5-kg/m^2^ group, were 0.772, 0.729, and 0.792, respectively (p < 0.001). Compared to those with normal color vision, participants with color weakness had an odds ratio of 0.956 for myopia development (p < 0.001), although color blindness was not significantly associated with myopia. Moreover, even though diabetes was not found to be a significant factor in univariable analysis, it was associated with an odds ratio of 1.044 (p = 0.004) for myopia development after adjustment for birth season, education level, BMI, and color vision (Table 2).

**Table 2:** Results of logistic regression analysis for the prevalence of myopia.

		%	Univariable			Multivariable		
			Odds ratio	95% CI	p-value	Odds ratio	95% CI	p-value
Birth season	Spring	56.93	1					
	Summer	56.20	0.971	(0.963–0.978)	< 0.001	0.976	(0.969–0.984)	< 0.001
	Autumn	56.17	0.969	(0.962–0.977)	< 0.001	0.971	(0.964–0.978)	< 0.001
	Winter	56.21	0.971	(0.964–0.978)	< 0.001	0.972	(0.964–0.979)	< 0.001
Education level	Less than high school	48.01	1					
	2–3-year university	51.36	1.143	(1.135–1.152)	< 0.001	1.148	(1.139–1.157)	< 0.001
	4-year university or higher	62.23	1.784	(1.773–1.796)	< 0.001	1.804	(1.792–1.816)	< 0.001
Height	< 170 cm	57.11	1					
	170–175 cm	56.40	0.972	(0.965–0.979)	< 0.001			
	175–180 cm	56.05	0.958	(0.951–0.965)	< 0.001			
	≥ 180 cm	55.68	0.944	(0.935–0.952)	< 0.001			
Weight	< 60 kg	59.04	1					
	60–66 kg	56.11	0.887	(0.880–0.894)	< 0.001			
	66–75 kg	55.09	0.851	(0.844–0.857)	< 0.001			
	≥ 75 kg	55.67	0.871	(0.865–0.878)	< 0.001			
BMI	< 18.5 kg/m^2^	61.25	1					
	18.5–23 kg/m^2^	56.07	0.808	(0.800–0.815)	< 0.001	0.772	(0.765–0.779)	< 0.001
	23–25 kg/m^2^	54.90	0.770	(0.762–0.779)	< 0.001	0.729	(0.721–0.737)	< 0.001
	≥ 25 kg/m^2^	56.04	0.806	(0.799–0.814)	< 0.001	0.792	(0.784–0.800)	< 0.001
Color vision	Normal	56.44	1					
	Color weakness	55.25	0.953	(0.941–0.965)	< 0.001	0.956	(0.944–0.968)	< 0.001
	Color blindness	54.73	0.933	(0.725–1.201)	0.592	0.958	(0.743–1.234)	0.738
Diabetes	No	56.39	1					
	Yes	55.84	0.978	(0.950–1.007)	0.141	1.044	(1.014–1.076)	0.004

BMI, body mass index. 95% CI, 95% confidence interval.

### Factors affecting high myopia

According to univariable logistic regression analysis, compared with spring-born individuals, those born in summer, fall, and winter had odds ratios of 0.975, 0.966, and 0.975, respectively, for the prevalence of high myopia (p < 0.001). The prevalence of high myopia was highest in spring, followed by summer and winter, and lowest when the birth season was autumn. The prevalence rate was higher in spring than in the other seasons.

Compared to high school graduates, participants with 2–3-year university and ≥ 4-year university education had odds ratios of 1.165 and 1.770, respectively, for myopia development (p < 0.001). The prevalence of high myopia was higher in men who attended 2–3-year colleges and ≥ 4-year colleges than in high school graduate men. The prevalence of high myopia was higher in men who attended 2–3-year colleges than in high school graduate men, but it was lower than that in men who attended 4-year colleges.

Compared to participants in the ≤ 170-cm group, participants in the 170–175- and 175–180-cm groups had odds ratios of 1.002 and 1.009, respectively, for high myopia; these associations were not significant (p = 0.711 and p = 0.074, respectively). There was a significant 1.037-fold increase in the odds ratio for hypermetropia for those with a height of > 180 cm (p < 0.001). The prevalence of high myopia was higher when the height was > 180 cm than when the height was ≤ 170 cm.

The odds ratios were 0.922, 0.908, and 0.977 for the 61–66, 67–75, and ≥ 76-kg groups, respectively, compared with the < 60-kg group (p < 0.001). The prevalence of high myopia was high when the weight was < 60 kg, and the prevalence was lower in the 66–75-kg group than in the other weight groups.

The odds ratios for the 18.5–22.99-, 23.00–24.99-, and 25-kg/m^2^ groups compared with the < 18.5-kg/m^2^ group were 0.844, 0.835, and 0.904, respectively (p < 0.001). The prevalence of high myopia was high when the BMI was < 18.5 kg/m^2^, whereas the prevalence of high myopia was low when the BMI was 23–25 kg/m^2^. The prevalence of high myopia was higher every year when height, weight, and BMI were > 180 cm, < 60 kg, and < 18.5 kg/m^2^, respectively. Compared to those with normal color vision, participants with color weakness and color blindness had odds ratios of 0.817 and 0.297, respectively, for high myopia development (both p < 0.001). Diabetes was not significantly associated with high myopia.

The results of multivariable logistic regression analysis showed the effect of each factor on high myopia after adjustment for other variables. The odds ratios for high myopia were 0.981, 0.968, and 0.976 when the birth season was summer, fall, and winter, respectively, compared to spring (p < 0.001). Compared to high school graduates, participants with 2–3-year college and ≥ 4-year college education had odds ratios of 1.169 and 1.790 for high myopia development, respectively (p < 0.001). Compared with the < 18.5-kg/m^2^ group, the greater BMI groups had odds ratios of 0.810, 0.795, and 0.892, respectively, for high myopia development (p < 0.001). The odds ratios for participants with color weakness and color blindness, compared to participants with normal color vision, were 0.818 and 0.297, respectively (p < 0.001). While diabetes was not found to be a significant factor in univariable analysis, it was associated with an odds ratio of 1.070 (p < 0.001) for high myopia development after adjustment for birth season, education level, BMI, and color vision (Table 3).

**Table 3:** Results of logistic regression analysis for the prevalence of high myopia.

		%	Univariable			Multivariable		
			Odds ratio	95% CI	p-value	Odds ratio	95% CI	p-value
Birth season	Spring	17.15	1			1		
	Summer	16.80	0.975	(0.966–0.985)	< 0.001	0.981	(0.971–0.991)	< 0.001
	Autumn	16.67	0.966	(0.956–0.975)	< 0.001	0.968	(0.959–0.978)	< 0.001
	Winter	16.79	0.975	(0.966–0.984)	< 0.001	0.976	(0.966–0.985)	< 0.001
Education level	Less than high school	12.39	1			1		
	2–3-year university	14.14	1.165	(1.152–1.178)	< 0.001	1.169	(1.156–1.183)	< 0.001
	4-year university or higher	19.99	1.770	(1.754–1.787)	< 0.001	1.790	(1.773–1.807)	< 0.001
Height	< 170 cm	16.74	1					
	170–175 cm	16.76	1.002	(0.993–1.011)	0.711			
	175–180 cm	16.87	1.009	(0.999–1.019)	0.074			
	≥ 180 cm	17.25	1.037	(1.025–1.049)	< 0.001			
Weight	< 60 kg	17.53	1					
	60–66 kg	16.39	0.922	(0.913–0.932)	< 0.001			
	66–75 kg	16.18	0.908	(0.899–0.917)	< 0.001			
	≥ 75 kg	17.21	0.977	(0.967–0.986)	< 0.001			
BMI	< 18.5 kg/m^2^	18.81	1			1		
	18.5–23 kg/m^2^	16.35	0.844	(0.834–0.854)	< 0.001	0.810	(0.800–0.820)	< 0.001
	23–25 kg/m^2^	16.21	0.835	(0.823–0.846)	< 0.001	0.795	(0.784–0.806)	< 0.001
	≥ 25 kg/m^2^	17.33	0.904	(0.893–0.916)	< 0.001	0.892	(0.881–0.903)	< 0.001
Color vision	Normal	16.98	1			1		
	Color weakness	14.33	0.817	(0.803–0.832)	< 0.001	0.818	(0.804–0.833)	< 0.001
	Color blindness	5.76	0.297	(0.174–0.509)	< 0.001	0.297	(0.173–0.508)	< 0.001
Diabetes	No	16.85	1			1		
	Yes	17.15	1.018	(0.980–1.058)	0.354	1.070	(1.030–1.112)	< 0.001

BMI, body mass index. 95% CI, 95% confidence interval.

## Discussion

### Main findings

This study showed that myopia and high myopia are increasing yearly in 19-year-old Korean men. However, the prevalence was lower in the present study than in a previous study in Korea^3^, probably because the MMA does not perform the test for autorefraction if the visual acuity is 0.3 or better. With this test method, the prevalence of myopia can actually be lower and, in fact, the prevalence of myopia can be higher. The prevalences of myopia and high myopia were high when the season of birth was spring and the educational level was high. Moreover, the prevalence of myopia was high when the height was ≤ 170 cm, weight was ≤ 60 kg, and BMI was < 18.5 kg/m^2^, while the prevalence of high myopia was high when the height was > 180 cm, weight was ≤ 60 kg, and BMI was < 18.5 kg/m^2^. The prevalences of both myopia and high myopia were higher when color vision was normal than when color weakness and color blindness were present, and when diabetes was present than when it was absent.

### Birth season

In China, as of September, the prevalence of myopia was lower when people were born later than earlier in the year^10^. Some authors have suggested an association between the age of onset of schooling and myopia. There is an age limit to enroll in school, with a difference of up to 1 year, and the progression of myopia tends to be faster at a young age than at an older age^21^. However, in Israel, although the school year starts in September^11^, individuals born in summer were found to have a higher prevalence of high myopia than did those born in other seasons.

In this study, 19-year-old men born in spring (March, April, and May) had a higher prevalence of myopia, while those born in fall (September, October, and November) had a lower prevalence. This suggests that the prevalence of myopia is associated with the time of admission; however, this cannot be certain. Myopia may be more strongly related to the season in each region than to the time of school admission. Therefore, further research is needed to determine if there is a relationship between the season of birth and myopia at school admission.

### Education and myopia

A study on Chinese individuals also found a relationship between education and myopia^10^. The prevalence of myopia and high myopia did not increase during the period of implementation of the easing education system in Japan^22^. Higher levels of school and after school education are associated with greater myopia refraction than lower levels. People with higher education levels more often have myopia than less educated individuals^7,9,23^. The longer the education period, the higher the prevalence of myopia^5,10,24^. Education and time spent outdoors are considered risk factors for myopia. There is consistent evidence suggesting a causal relationship between more education and myopia. Close-range work may contribute to myopia, but this is not clear. This can be considered a mediating factor as it affects other factors^21^.

Compared with the results of a previous study, the prevalence of myopia increased at all levels, but among them, the prevalence of myopia increased substantially at the 2–3-year university education level in the present study. This may be attributed to the fact that people may attend a 2–3-year university for the purpose of seeking employment rather than a 4-year university or higher. In addition, the use of a smartphone, tablet, and laptops in Korea’s educational and daily environments is increasing. The number of students studying using tablets even in their teens is also increasing, and computers or monitors are also being used in classes. Therefore, more students may have myopia than before.

Moreover, the students who retake classes were included in the high school graduate group because they did not attend a university. However, the recent trend is that the rate of attending a university with high school grades is increasing, the number of repeat students is decreasing, and the rate of attending 2–3-year universities and 4-year universities or higher is increasing. Because of this increase, there may be some differences between our study and previous studies’ findings.

### Color vision and diabetes

Studies have shown that the rate of myopia is low in the case of color weakness and color blindness^9,17–19^. The results are consistent with those of a previous study, which reported lower myopia development in individuals with color weakness and color blindness^18^. Another study reported that the risk of myopia is high in individuals with diabetes^20^. In contrast, no association between diabetes and myopia has been reported^12^. Therefore, additional research is needed to determine whether color blindness and diabetes are associated with myopia.

Diabetes mellitus is a disease characterized by a high blood glucose concentration due to insufficient insulin secretion or failure of the pancreas to function normally. When the concentration of glucose in the blood is high, the concentration of the aqueous humor in the eye increases, and a large amount of glucose enters the lens. Simultaneously, excess water enters the lens. Therefore, when the blood glucose level is high, the lens becomes thicker than usual, and the refractive power increases owing to the thickened lens, leading to myopia.

The present study may serve as a stepping stone for future research, as not many studies have investigated whether color vision and diabetes are related to myopia. In particular, in the case of diabetes, there seemed to be no association with myopia in the univariable analysis, but there seemed to be a significant association after adjusting for other variables. Therefore, examining the relationship between diabetes and myopia would be interesting.

### Height, weight, and the BMI

One study found a significant association with myopia when height, weight, and BMI were high^25^; another study reported that the prevalence of myopia was low when BMI was normal, whereas it was high when BMI was low^26^. In a previous study, the prevalence of myopia was also high when BMI was low^6^. However, in other studies on Koreans, height, weight, and BMI were not related to myopia^3,9^. Herein, the prevalences of myopia and high myopia were high when BMI was low. The prevalence of myopia varies according to height, weight, and BMI. Previous studies have reported that height and axial length were positively correlated, and that myopia varied according to the axial length^27,28^. In this study, the prevalence of myopia decreased with height, but the prevalence of hyper myopia increased significantly only in those who were 180 cm or taller, relative to that in individuals with a height of < 170 cm. These findings indicate that high myopia was more strongly correlated with the axial length than was myopia, as reported in a previous study^6^. These conflicting results for myopia and high myopia, as well as those of previous studies, make it difficult to draw clear conclusions. Using those studies to determine the overall prevalence and trends in a meta-analysis is also recommended.

### Limitations

The limitation of this study is that only 19-year-old male participants were included, and the educational level categories were limited to high school graduates and university students. Since only personal physical examination information was used, parental information (genetic information) could not be obtained, and only the myopic eye was used as the sample. Additionally, as this was a retrospective study, we could not obtain all necessary data or variables. Some data were not available in raw form, and diabetes was diagnosed on the basis of a single diagnostic test. Therefore, we could not specify the criteria for some variables, such as diabetes and color vision. Furthermore, it is impossible to know whether refractive surgery had been performed before the physical examination. Nowadays, many individuals in Korea undergo refractive surgery, which is negatively associated with myopia. In addition, color vision and diabetes were found to be risk factors for myopia only in the multivariate analysis in this study, but there was an imbalance in the number of participants depending on the presence or absence of myopia. Therefore, a more accurate conclusion can be reached if follow-up studies include both a diabetic and a color vision group, in which the number of participants will be balanced.

In conclusion, when the birth season was spring, myopia and high myopia were high. A higher level of education was associated with a higher prevalence of myopia and high myopia. The prevalence of myopia was high when the height was short, weight was low, and the BMI was low. In particular, height as a risk factor was the opposite for myopia and high myopia. When color vision was normal, the prevalences of myopia and high myopia were high. In the case of color blindness, there was a significant difference only in high myopia. Diabetes did not appear to affect myopia and high myopia as a single variable, but after adjusting for other factors (season of birth, BMI, and color blindness), the prevalences of myopia and high myopia were higher in the presence of diabetes than in its absence.

## Data Availability

The data that support the findings of this study are available from Military Manpower Administration of the Republic of Korea, but restrictions apply to the availability of these data, which were used under license for the current study, and so are not publicly available. Data are however available from the authors upon reasonable request and with permission of Military Manpower Administration of the Republic of Korea.
